# Radar-Based Microwave Breast Imaging Using Neurocomputational Models

**DOI:** 10.3390/diagnostics13050930

**Published:** 2023-03-01

**Authors:** Mustafa Berkan Bicer

**Affiliations:** Electrical and Electronics Engineering Department, Engineering Faculty, Tarsus University, 33400 Mersin, Turkey; mberkanbicer@tarsus.edu.tr

**Keywords:** inverse scattering, circular synthetic aperture radar (CSAR), breast imaging, deep neural networks (DNNs), convolutional neural networks (CNNs)

## Abstract

In this study, neurocomputational models are proposed for the acquisition of radar-based microwave images of breast tumors using deep neural networks (DNNs) and convolutional neural networks (CNNs). The circular synthetic aperture radar (CSAR) technique for radar-based microwave imaging (MWI) was utilized to generate 1000 numerical simulations for randomly generated scenarios. The scenarios contain information such as the number, size, and location of tumors for each simulation. Then, a dataset of 1000 distinct simulations with complex values based on the scenarios was built. Consequently, a real-valued DNN (RV-DNN) with five hidden layers, a real-valued CNN (RV-CNN) with seven convolutional layers, and a real-valued combined model (RV-MWINet) consisting of CNN and U-Net sub-models were built and trained to generate the radar-based microwave images. While the proposed RV-DNN, RV-CNN, and RV-MWINet models are real-valued, the MWINet model is restructured with complex-valued layers (CV-MWINet), resulting in a total of four models. For the RV-DNN model, the training and test errors in terms of mean squared error (MSE) are found to be 103.400 and 96.395, respectively, whereas for the RV-CNN model, the training and test errors are obtained to be 45.283 and 153.818. Due to the fact that the RV-MWINet model is a combined U-Net model, the accuracy metric is analyzed. The proposed RV-MWINet model has training and testing accuracy of 0.9135 and 0.8635, whereas the CV-MWINet model has training and testing accuracy of 0.991 and 1.000, respectively. The peak signal-to-noise ratio (PSNR), universal quality index (UQI), and structural similarity index (SSIM) metrics were also evaluated for the images generated by the proposed neurocomputational models. The generated images demonstrate that the proposed neurocomputational models can be successfully utilized for radar-based microwave imaging, especially for breast imaging.

## 1. Introduction

In the health care industry, the diagnosis and treatment of diseases has become increasingly reliant on rapidly advancing technology. Currently, cardiovascular diseases are the leading cause of death, followed by cancer in second place [1,2]. Although cancer is a non-communicable disease with various types, breast cancer is the most prevalent form of cancer among women [1,3]. Despite the fact that breast cancer can be discovered reasonably quickly and easily due to the development of medical imaging technologies, if it is not diagnosed at an early stage, it can develop into later stages and be fatal. In addition, it is crucial to detect breast cancer at an early stage since it might metastasize and spread to other tissues, resulting in the development of additional malignancies. Although a variety of modalities are used to identify breast cancer at an early stage, X-ray mammography is the most frequently utilized primary modality [3]. However, the drawbacks of X-ray mammography include the use of ionizing X-rays for imaging, low mobility, low sensitivity, and painful compression of breast tissue between two planes. In addition, X-ray mammography, which is significantly more effective in detecting benign cancers, may necessitate an additional biopsy to detect malignant tumors [3]. Ultrasonography (USG), which is used as an adjunct to X-ray mammography, utilizes sound waves for imaging purposes, at frequencies inaudible to the human ear. Since the penetration depth of the sound waves into the human body is not particularly deep, it is important to apply pressure to the body with the probe and use a matching medium for good imaging, despite the fact that these sound waves convey information about the breast tissue. In addition, when a mass is found by USG, a biopsy should be performed to obtain further information about the mass. Magnetic resonance imaging (MRI) has become an alternative to X-ray mammography and USG by generating images based on the principle of magnetic resonance. The imaging provided by measuring the response of the body of the patient, which has been subjected to a magnetic field, to some waves applied in this area has a higher sensitivity than other techniques but a lower specificity. In addition, MRI has drawbacks such as a high cost, a longer imaging procedure, and an unpleasant measurement. These drawbacks of primary modalities such as X-ray mammography, USG, and MRI have motivated researchers to develop alternative techniques. Microwave imaging (MWI) is an alternative imaging modality that utilizes low-frequency and low-power electromagnetic waves for imaging and has been intensively researched by researchers. In MWI, electromagnetic waves in the non-ionizing microwave frequency band are generated and used to illuminate breast tissue with electromagnetic waves through antennas. MWI offers significant advantages over conventional modalities, thanks to its specially designed measurement instruments that may provide a more comfortable examination. In addition, electromagnetic waves can be generated via cost-effective and mobile MWI devices, and systems that are easily transportable to regions where mobility is required can be constructed. Researchers in the field of MWI have conducted numerous studies, particularly concerning the operating frequency and imaging methods [4,5,6,7,8,9,10,11,12,13,14,15,16,17,18,19,20,21,22,23,24]. Li et al. [20] proposed a CNN-based model for solving non-linear inverse electromagnetic problems with deep learning (DL) models. The images were obtained by collecting the scattered electromagnetic fields from the illuminated target and applying these collected fields to the DL model. The authors [20] discuss the theory underlying the relationship between DL models and non-linear inverse electromagnetic problems, and demonstrate the performance of their approach using the Modified National Institute of Standards and Technology (MNIST) dataset. Barrachina et al. [25] proposed the use of complex-valued and real-valued U-Net models for semantic segmentation in polarimetric synthetic aperture radar (PolSAR) images. Jing et al. [26] presented a CNN model with complex values for near-field millimeter-wave imaging. The proposed model consists of fully conventional layers and enhances the input image data. Experimental measurements were conducted at 34.5 GHz, and the performance of the model was demonstrated using the measurement results. Yadav et al. [27] developed a microwave tomography (MWT) approach based on neural networks for usage in industrial microwave drying systems. The authors intended to determine the distribution of moisture in an industrial drying system using this method. In their study, experiments were performed utilizing a linear MWT array to determine the distribution of moisture in the Hephaistos microwave oven system. Wang et al. [5] proposed a compressed-sensing (CS)-based convolutional neural network (CSR-Net) model for microwave sparse reconstruction. The authors validated the performance of their proposed model on various simulated and measured data. The authors also performed three-dimensional imaging using the results of the model they developed using complex-valued data. Ambrosanio et al. [28] proposed a deep neural network model for breast imaging. The model estimates the dielectric constant and tissue conductivity using the scattered electric field matrix as input data. The performance of the model, which comprises 3 layers with 2000 nodes each, is compared to the cross-correlated contrast source inversion (CC-CSI) [29] and adaptive multi-threshold iterative shrinkage thresholding algorithm (AMTISTA) [30] techniques. Dey et al. [21] presented an approach for breast lesion localization in microwave imaging utilizing pulse-coupled neural networks (PCNN). The authors of reference [21] obtained 61 breast images of 35 individuals using microwave imaging from a matching-liquid-free system operating between 1 GHz and 9 GHz, and 81.82% success was achieved as a malignant finding (MF) performance. Shao et al. [31] developed an auto-encoder-based DL algorithm that transforms 4 GHz data received from 24 × 24 antenna array data into 128 × 128 images. The performance of the model was evaluated by comparing the images using the distorted-Born iterative method (DBIM) and the phase confocal method (PCM) techniques. The developed model [31] utilizes the complex input data as a two-dimensional image in amplitude and phase. Chiu et al. [32] examined the U-Net and object-attentional super-resolution network (OASRN) models for electromagnetic imaging. Using a setup of 32 transmitting and 32 receiving antennas, scattered field measurements were carried out with the addition of Gaussian noise. The authors [32] concluded that the OASRN model is superior to the U-Net model based on a comparison of the obtained images and results. Khoshdel et al. [22] developed a model based on DL for three-dimensional breast imaging. Three-dimensional CSI images are applied as the input to the proposed U-Net-based DL model, and a three-dimensional dielectric map is generated as output. It has been demonstrated that the U-Net model, which enhances the CSI images applied to the input, produces superior results as compared to the CSI method [22]. Qin et al. [23] developed a breast imaging model based on DL using microwave and ultrasonic data. The proposed model [23] utilizes ultrasound and microwave data as input, combines them, and applies convolutional layers. The output of the model is divided into two branches to provide the segmentation result and regression results, such as the dielectric constant. Considering the studies in the literature [22,33,34,35,36], it can be seen that the application of DL models in medical imaging systems is rising. DL models produce faster and higher-quality results than conventional imaging techniques, and they are becoming more popular in imaging systems.

In this study, four models utilizing deep neural networks and convolutional neural networks are proposed for the generation of monostatic radar-based microwave images using backscattered electric field data using the CSAR principle. The images generated by the models are compared to those obtained by a matching pursuit-based (MP-based) [19,37] algorithm, and the performances of the models are discussed.

The highlights of this study are as follows:

- In this study, conventional imaging was carried out utilizing CSAR-based numerical data and an MP-based algorithm.
- For imaging, both the matching-pursuit-based method and the neurocomputational models utilized raw, unprocessed real-valued, and complex-valued numerical data. Computed or measured scattered electric field data can therefore be applied directly to models without preprocessing.
- RV-DNN and RV-CNN models are proposed, followed by two combined neurocomputational models (RV-MWINet and CV-MWINet) employing the proposed CNN model structure, which combines the U-Net structure. The images generated by the proposed models are compared to those generated by the matching-pursuit algorithm. The study demonstrates that the processing and generation speeds of the proposed models are faster than those of conventional imaging techniques, and that the resulting images are of higher quality.
- By placing a screw in the sand and an unhealthy tumor phantom in a healthy phantom, a total of 12 measurements were taken in the range of 1 GHz to 10 GHz, using the measurement setup. In order to train the CV-MWINet model, measurement data were added to the dataset obtained from simulated data. Also, the performance of the proposed model on both simulated and measured data is discussed.

## 2. The Forward Problem Based on the Circular Synthetic Aperture Radar (CSAR) Principle

**Figure 1:**
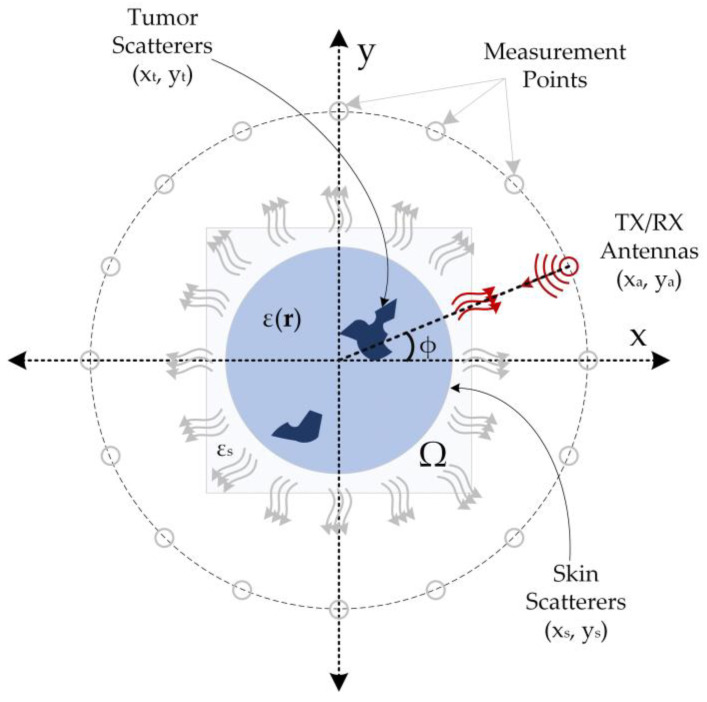
Simulation setup for two-dimensional breast tumor imaging (The red arcs from the antenna to the imaging field represent the propagating wave, the gray arrows the scattered field, and the red arrows the backscattered field).

The simulation data used in this study were generated based on the monostatic circular synthetic aperture radar (CSAR) principle [38], and the simulation data acquisition setup is illustrated in Figure 1. In this method, a transceiver antenna is rotated at certain intervals on a concentric circle with a stationary object in the imaging domain (Ω) with a dielectric distribution ε(**r**), and collects backscattered electric field data from this domain. This method assumes that the imaging domain is entirely encompassed by the radiation pattern of the antenna. Thus, the electric field measurements backscattered from the imaging domain contain information about the target object. The backscattered electric field data obtained in accordance with the structure depicted in Figure 1 comprise information regarding skin and tumors.

According to the CSAR concept, the back-scattered electric field in frequency domain can be expressed as [37],

(1) $$E_{s}\left(f,\phi \right)=A_{0}\cdot e^{-j\cdot \frac{4\pi f\sqrt{\varepsilon_{r}\mu_{r}}}{c}\cdot R\left(\phi \right)},$$

where *A*_0_, *f*, *ε_r_*, *μ_r_*, *c*, and ***R***(*ϕ*) denote the amplitude of the electric field, frequency, relative permittivity, magnetic permeability, the phase velocity of the wave, and the Euclidean distance function between the scatterer and antenna. For most common materials, *μ_r_* is considered as 1. For the sake of simplicity, the imaging field is considered to be homogenous, and the tumor and skin are supposed to be discrete perfect scatterers. The angle-dependent Euclidean distance in the expression given in Equation (1) is calculated by Equation (2) [38].

(2) $$R\left(\phi \right)=\sqrt{{\left|x_{a}-R_{0}\cdot \cos\left(\phi \right)\right|}^{2}+{\left|y_{a}-R_{0}\cdot \sin\left(\phi \right)\right|}^{2}}$$

As shown by the equation, the distance is calculated using the difference between the antenna position and the projection of the scatterers on the axis. The single transceiver antenna in the imaging system collects the backscattered electric field data from the imaging domain by positioning itself at the measurement positions shown in Figure 1 at predetermined intervals. For each measurement point, the backscattered electric field data from all scattering points within the imaging domain is collected to yield the overall electric field data. This procedure is repeated for all measurement points, resulting in 360-degree data coverage of the imaging region. The measured data may contain information regarding the maximum range (*R_m_*), which can be determined using Equation (3) [38].

(3) $$R_{m}=N\cdot \Delta r,$$

where *N* represents the number of frequencies, Δ*r* represents the range resolution and is calculated using Equation (4) [38].

(4) $$\Delta r=\frac{c}{2\cdot N\cdot \Delta f}$$

Δ*f* in Equation (4) represents the bandwidth used in the measurement system. The parameters and values specified in Table 1 were utilized to acquire the total backscattered electric field data from the imaging plane.

Using Equation (1) through (4), between one and three tumor scatterers with diameters between 0.2 cm and 0.9 cm and random positions and shapes in the imaging domain were generated, and numerical data for these scatterers were computed. Consequently, a complex-valued backscattered electric field dataset for 1000 scatterers was created. The dataset, each consisting of backscattered electric field data with dimensions (301 × 90), had dimensions (1000, 301, 90) in total (number of data, number of frequencies, number of angles).

## 3. Phantom Fabrication and Measurement

**Figure 2:**
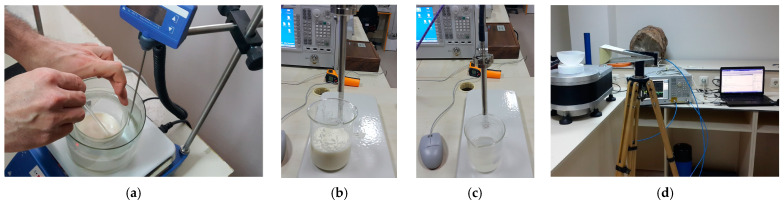
(**a**) Fantom fabrication, dielectric constant measurement of (**b**) healthy and (**c**) tumor phantoms and (**d**) microwave measurement setup for two-dimensional breast tumor imaging.

In this study, measurements were carried out to be used for model training. To obtain the measurement data, phantoms of both healthy and tumor tissues were fabricated using methods similar to those described by Ortega-Palacios et al. [39]. Figure 2 depicts the images of the phantom fabrication, dielectric constant measurements, and microwave imaging measurement setup.

**Figure 3:**
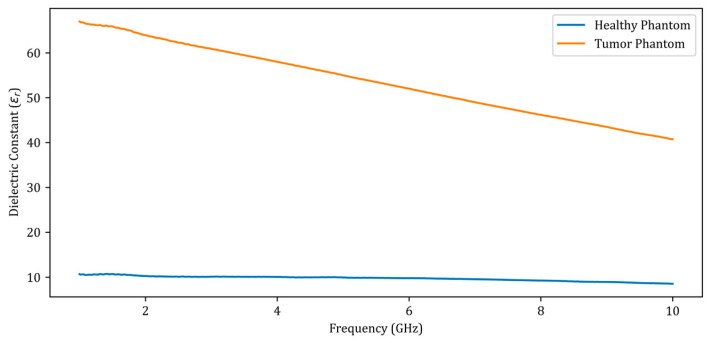
Dielectric constants of the fabricated phantoms between 1 GHz and 10 GHz.

Using a dielectric probe, the dielectric constants of the phantoms were measured between 1 GHz and 10 GHz, as shown in Figure 2a. Figure 2b depicts a measurement setup in a large, empty space outside the setup. During the measurements, an ultra-wideband (UWB) horn antenna was employed. For the sake of simplicity, the rotation of the material was chosen over the antenna in the measurement setup. The computer-controlled turntable was rotated at angles of 4 degrees, and the scattering parameter (S_11_) was measured at a total of 90 angles for a total of 360 degrees. Figure 3 depicts the dielectric constant measurement graph of the phantoms manufactured as shown in Figure 2a.

When analyzing the dielectric constants presented in Figure 3 for the healthy phantom and the tumor phantom, a dielectric contrast of 4 to 6 is observed. There was a total of 12 measurements performed, including 7 obtained by placing metal screws at 7 distinct locations in the fine sand and 5 obtained by placing the tumor phantom at 5 points on the healthy phantom. The measurement data were added to the dataset used to train the deep learning model along with the simulation data.

## 4. Microwave Imaging (MWI) Using Deep Learning (DL) Models

The similarities between DL models and non-linear electromagnetic scattering are initially discussed in this study. Then, the use of three distinct real-valued and one complex-valued DL approaches will be explained. These are real-valued deep neural network-based (RV-DNN), real-valued convolutional network-based (RV-CNN), and combined real-valued and complex-valued DL models consisting of CNN and U-Net-based models (RV-MWINet and CV-MWINet).

### 4.1. Similarities between DL and Non-Linear Electromagnetic Scattering

The relationship between DL and non-linear electromagnetic scattering, as established by Li et al. [20], is considered in this study. For the configuration depicted in Figure 1, the total electric field value $E^{\left(n\right)}\left(r\right)$, where $E_{i}^{\left(n\right)}\left(r\right)$ is the total incident electric field and $E_{s}^{\left(n\right)}$ is the total scattered electric field, can be calculated using Equation (5) [20].

(5) $$\begin{array}{cc} E^{\left(n\right)}\left(r\right) & =E_{i}^{\left(n\right)}\left(r\right)+E_{s}^{\left(n\right)}\\ & =E_{i}^{\left(n\right)}\left(r\right)+k_{0}^{2}\int_{\Omega }\left(\frac{i}{4}\right)H_{0}^{\left(1\right)}\left(\left|r-r^{\prime }\right|\right)\chi \left(r^{\prime }\right)E^{\left(n\right)}\left(r^{\prime }\right)dr^{\prime } \end{array}$$

The parameters *n*, *k*_0_, $H_{0}^{\left(1\right)}$ and χ represent the index of the scattering, the wavenumber of the background medium, the first-kind zeroth-order Hankel function, and the contrast function, respectively. ***r*** = (x, y) and ***r***’ = (x’, y’) indicate the field and source positions, respectively, and are evaluated as ***r***, ***r***’ ∈ Ω. In computational imaging, the imaging region surrounded by antennas and whose content is unknown is regarded as being divided into pixels. The values of the pixels provide information related to the contrast values. Consequently, the value of the scattered electric field to be used in the imaging process is computed using Equation (6) [20].

(6) $$E_{s}^{\left(n\right)}=G_{d}E^{\left(n\right)}\chi$$

(7) $$E^{\left(n\right)}-E_{inc}^{\left(n\right)}=G_{s}E^{\left(n\right)}\chi$$

Green’s function is represented by **G** in Equations (6) and (7). Iteratively applying Equations (6) and (7) yields the expression given in Equation (8) for the (*t*+1)th stage of the contrast function [20].

(8) $$\chi_{\left(t+1\right)}=\arg\underset{\chi }{\min}\left[{\sum_{n}\left\|\delta E_{s}^{\left(n\right)}-J_{\left(t\right)}^{\left(n\right)}\delta \chi \right\|}_{2}^{2}+ℜ\left(\chi \right)\right]$$

In Equation (8), $\delta E_{s}^{\left(n\right)}$ and δχ are defined as $\delta E_{s}^{\left(n\right)}\equiv E_{s}^{\left(n\right)}-E_{s}^{\left(n\right)}\left(\chi_{\left(t\right)}\right)$ and $\delta \chi \equiv \chi -\chi_{\left(t\right)}$. The (*t*) indices in the expressions denote the value of *t*-th iteration. $J_{\left(t\right)}^{\left(n\right)}$ represents the Jacobian matrix of $E_{s}^{\left(n\right)}$ with regard to $\chi_{\left(t\right)}$. ℜ(χ), which denotes the regularization in Equation (8), is defined as shown in Equation (9) for simplicity [20].

(9) $$ℜ\left(\chi \right)={\left\|D\chi \right\|}_{1}$$

In Equation (9), the parameter **D** is utilized to describe a sparse transformation process like a wavelet. The contrast function at time *t* + 1 can be defined as in Equation (10) [20].

(10) $$\chi_{\left(t+1\right)}=D^{H}S\left\{D\chi_{\left(t\right)}+D{\left[\sum_{n}{\left(J_{\left(t\right)}^{\left(n\right)}\right)}^{H}J_{\left(t\right)}^{\left(n\right)}\right]}^{†}\sum_{n}{\left(J_{\left(t\right)}^{\left(n\right)}\right)}^{H}\delta E_{s}^{\left(n\right)}\right\}$$

*S*{.} and *H* in Equation (10) denote the element-wise soft-threshold and conjugate transpose, respectively. Equation (10) can be rearranged as Equations (11) and (12) to illustrate the connection between NN and non-linear electromagnetic scattering [20].

(11) $$\begin{array}{cc} D\chi_{\left(t+1\right)} & =S\left\{D\chi_{\left(t\right)}+D{\left[\sum_{n}{\left(J_{\left(t\right)}^{\left(n\right)}\right)}^{H}J_{\left(t\right)}^{\left(n\right)}\right]}^{†}\sum_{n}A_{\left(t\right)}^{\left(n\right)}\delta E_{s}^{\left(n\right)}\right\}\\ & =S\left\{D\chi_{\left(t\right)}+D{\left[\sum_{n}{\left(J_{\left(t\right)}^{\left(n\right)}\right)}^{H}J_{\left(t\right)}^{\left(n\right)}\right]}^{†}\times \sum_{n}A_{\left(t\right)}^{\left(n\right)}\left(E_{s}^{\left(n\right)}-G_{d}E_{\left(t\right)}^{\left(n\right)}\chi_{\left(t\right)}\right)\right\} \end{array}$$

(12) $$D\chi_{\left(t+1\right)}=S\left\{P_{\left(t\right)}\chi_{\left(t\right)}+b_{\left(t\right)}\right\}z$$

The parameters **P**_(t)_ and **b**_(t)_ in Equation (11) are given in Equations (13) and (14) [20].

(13) $$P_{\left(t\right)}\equiv DP_{\left(t\right)}-DP_{\left(t\right)}{\left[\sum_{n}{\left(J_{\left(t\right)}^{\left(n\right)}\right)}^{H}J_{\left(t\right)}^{\left(n\right)}\right]}^{†}\sum_{n}A_{\left(t\right)}^{\left(n\right)}G_{d}E_{\left(t\right)}^{\left(n\right)}$$

(14) $$b_{\left(t\right)}\equiv D{\left[\sum_{n}{\left(J_{\left(t\right)}^{\left(n\right)}\right)}^{H}J_{\left(t\right)}^{\left(n\right)}\right]}^{†}\sum_{n}A_{\left(t\right)}^{\left(n\right)}E_{s}^{\left(n\right)}$$

Equation (12) is conceptually comparable to the definition of a fully connected NN. The parameters **P** and **b** in Equation (12) correspond to the weights and bias values of fully connected NNs. The indices (*t*) of these parameters represent the neural network layers. This similarity and relationship demonstrate that DL models are applicable to non-linear electromagnetic scattering challenges.

### 4.2. Deep Neural Network-Based (DNN-Based) Imaging

**Figure 4:**
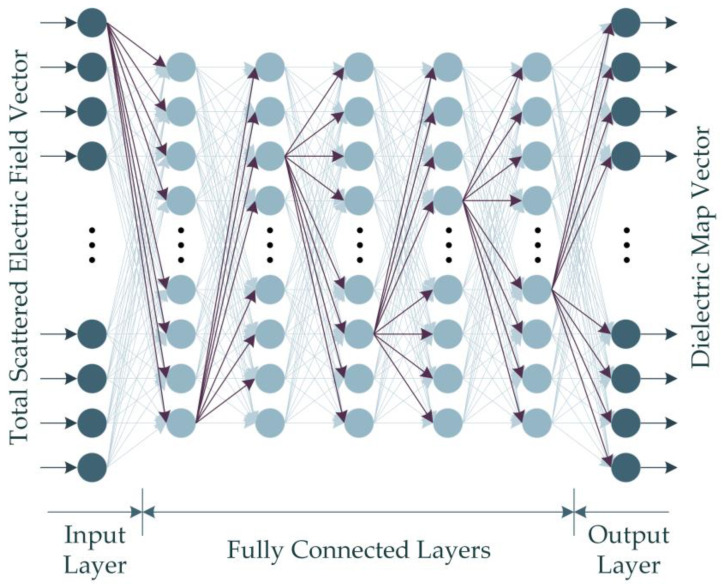
The proposed RV-DNN model for microwave medical imaging.

Given that the dataset built through numerical computations in this study contains backscattered electric field data with complex values, the real-valued DNN (RV-DNN) model is constructed to handle the absolute value of the complex values. Figure 4 illustrates the representative architecture of the proposed RV-DNN model.

The input values supplied to the model at the input layer are passed straight to the first layer by the input elements depicted in Figure 4. Using Equation (15), the outputs of each element in the hidden layers and the output layer are computed.

(15) $$h_{i}=\sigma \left(\sum_{j=1}^{N}W_{ij}x_{j}+b_{i}^{h}\right)$$

In Equation (15), the parameters *h_i_*, *N*, *W_ij_*, *x_j_*, and *b_i_^h^* represent the output value of the element, the number of inputs to the element, the weight coefficients at the input of the element, the values at the input of the element, and the bias value, respectively. The parameter *σ* represents the activation function, and the rectified linear unit (ReLU) activation function used in this study is given in Equation (16).

(16) σ=max(0, u)

Each of the 1000 data in the dataset comprises magnitude values of the complex-valued backscattered electric field data with a size of (301 × 90). The RV-DNN model was constructed to handle real-valued input and output data in one dimension. Thus, the two-dimensional input and output data were transformed into one-dimensional vectors, and the model was trained using these vectors. The model, which is designed with an input layer consisting of 27,090 elements, contains a total of 5 hidden layers, with the number of elements being (128, 128, 128, 128, 128). The output layer of the model comprises 16,384 elements, as the size of the image to be generated using the model is 128 × 128. The chosen settings for the training phase of the model include using the ReLU function as the activation function, the Adam algorithm as the optimization technique, an epoch number of 1000, a batch size of 32, and minimizing the mean squared error (MSE) as the metric. The 10-fold cross-validation method was applied to evaluate the performance of the model. In addition to cross-validation, the model was trained with 90% data and tested with 10% data. To compare the performance of the models considered in the study, images were also obtained using the traditional MP-based imaging algorithm.

### 4.3. Convolutional Neural Networks-Based (CNN-Based) Imaging

In this study, a sequential real-valued CNN (RV-CNN) model for the imaging of the backscattered electric field data is proposed. In the convolution process, the filtered output data are obtained by convolving the input data with the filter, also known as the kernel matrix. The filtering allows for the extraction of various attributes of the handled data. The convolution of the input data *x* with the four-dimensional *f* filter is calculated using Equation (17) [40]. The *x^l^*^+1^ derived from Equation (17) belongs to the solution set ${\mathbb{R}}^{H^{l+1}\times W^{l+1}\times D^{l+1}}$.

(17) $$x_{i^{l+1},j^{l+1},d}^{l+1}=\rho \left(\sum_{i=0}^{H}\sum_{j=0}^{W}\sum_{d^{l}=0}^{D^{l}}f_{i,j,d^{l},d}\times x_{i^{l+1}+i,j^{l+1}+j,d^{l}}^{l}\right)$$

In the equation, *x^l^* represents the input of the *l*th layer, while *x^l^*^+1^ represents the output of this layer, as well as the input of the (*l* + 1)th layer. *f* represents the kernel function for ${\mathbb{R}}^{H\times W\times D^{l}\times D}$, while the ρ function is the activation function.

**Figure 5:**
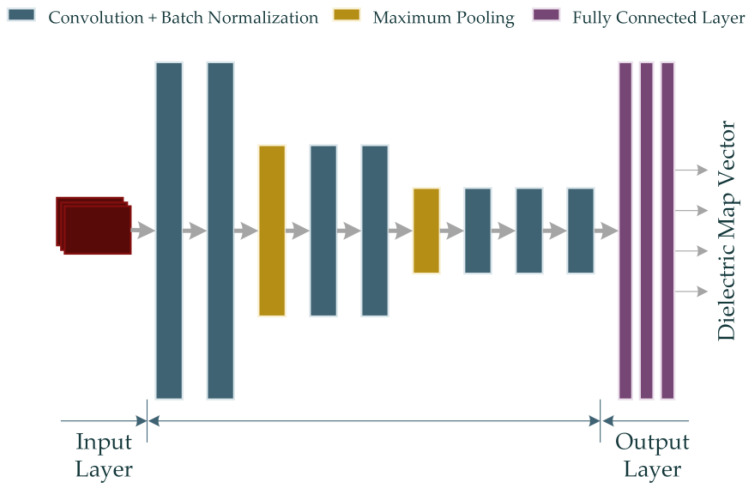
The proposed RV-CNN model for microwave medical imaging (Input data is shown in red color).

Also, the rest of the parameters are defined as *H^l^*^+1^ = *H^l^ – H* + 1, *W^l^*^+1^ = *W^l^ − W* + 1 and *D^l^*^+1^ = *D*. *H* × *W* represents the spatial span of each kernel, whereas *D* indicates the total number of kernels. The RV-CNN model developed in this study also employs the ReLU activation function derived from Equation (16). The RV-CNN model proposed in the study is shown in Figure 5.

The model shown in Figure 5 contains 7 convolutions and 3 fully connected layers. Details of the properties of the layers are given in Table 2.

As with the proposed RV-DNN model, the RV-CNN model is designed to obtain the one-dimensional dielectric map vector. In order to train the model, 1000 input data consisting of (301 × 90) backscattered electric field values were utilized. At the output of the model, a total of 1000 data consisting of one-dimensional dielectric map vectors of length 16,384 were obtained through training. The output vector is reshaped into a two-dimensional form during the imaging step. The proposed RV-CNN model was trained using the ReLU function as the activation function, Adam algorithm as the optimization algorithm, 2000 epochs, a batch size of 32, and the mean squared error (MSE) as the metric to minimize. Similar to the RV-DNN model, 10-fold cross-validation approach was used to evaluate the performance of the model.

### 4.4. U-Net-Based Combined Neurocomputational Imaging Model

**Figure 6:**
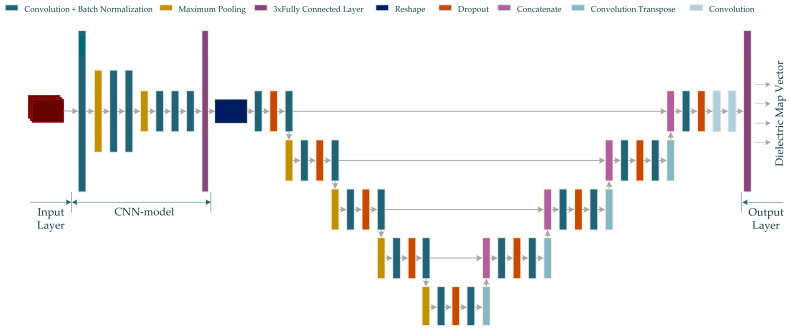
The proposed MWINet model for microwave medical imaging (Input data is shown in red color).

In this study, two neurocomputational models, named MWINet, are proposed for use in microwave imaging by combining the proposed CNN model with the U-Net-based model. For this purpose, a U-Net-based model extends the sequential CNN model. The proposed model utilizes raw scattered electric field data as the input and generates a one-dimensional microwave image. The structure of the proposed MWINet model is given in Figure 6.

As seen from Figure 6, the CNN structure in the initial layers of the proposed MWINet model provides general imaging, while the U-Net section is responsible for image cleaning and tumor structural clarification. For the purposes of this study, the layers of the model depicted in Figure 6 were constructed as RV-MWINet models with real-valued layers and CV-MWINet models with complex-valued layers.

In order to train the RV-MWINet model, 1000 input data consisting of (301 × 90) backscattered electric field values were utilized. At the output of the model, a total of 1000 data consisting of one-dimensional dielectric map vectors of length 16,384 were obtained through training. The output data used to train the model was converted to be binary valued. The output vector is reshaped into a two-dimensional form during the imaging step.

For the proposed RV-MWINet model, the real-valued ReLU function was chosen as the activation function for the inner layers, and the sigmoid activation function was chosen for the output layer. In the layers of the CV-MWINet model; however, the cartesian ReLU (CReLU) activation function given by Equation (18) is used, but the amplitude of the complex sigmoid function given by Equation (19) is used in the output layer.

(18) σCReLU=max(0,x)+jmax(0,y)

(19) $$\sigma_{CSigmoid}=\frac{1}{1+e^{-x}}+j\frac{1}{1+e^{-y}}$$

In Equations (18) and (19), the parameters *x* and *y* represent the real and imaginary components of the input data, respectively. The optimization algorithm selected was Adam, with 500 epochs, a batch size of 32, and accuracy as the metric to be maximized. Similar to the proposed RV-DNN and RV-CNN models, a 10-fold cross-validation approach was used to evaluate the performance of the model. While 1000 real-valued data were used to train and evaluate the performance of the RV-MWINet model, 12 measurement data were added to the data used to train and analyze the performance of the CV-MWINet model.

### 4.5. Evaluation Metrics

In this study, accuracy (*ACC*), mean squared error (*MSE*), peak signal-to-noise ratio (*PSNR*), universal quality image index (*UQI*), and structural similarity (*SSIM*) metrics were utilized to examine the images generated by the proposed neurocomputational models. For the *MSE* metric, the equation given in Equation (20) is used.

(20) $$MSE=\frac{1}{mn}\sum_{i=0}^{m-1}\sum_{j=0}^{n-1}{\left[x\left(i,j\right)-y\left(i,j\right)\right]}^{2}$$

The variables *x* and *y* in the equation represent the input and output images of size *m* × *n*. Although *MSE* is a significant metric in regression problems, it is more typical to utilize the well-known *PSNR*, *UQI*, and *SSIM* metrics to visually analyze images. Equation (21) is utilized to calculate the *PSNR* measure.

(21) $$PSNR=10\cdot \log_{10}\left(\frac{M_{I}^{2}}{MSE}\right)$$

The *M_I_* parameter in the equation represents the maximum value of the pixels. In addition to *PSNR*, the *UQI* and *SSIM* metrics given in Equations (27) and (28) provide significant information about the generated images. The values of the variables used in Equations (27) and (28) are calculated by Equations (22)–(26).

(22) $$\mu_{x}=\frac{1}{N}\sum_{i=1}^{N}x_{i}$$

(23) $$\mu_{y}=\frac{1}{N}\sum_{i=1}^{N}y_{i}$$

(24) $$\sigma_{x}^{2}=\frac{1}{N-1}\sum_{i=1}^{N}{\left(x_{i}-\mu_{x}\right)}^{2}$$

(25) $$\sigma_{y}^{2}=\frac{1}{N-1}\sum_{i=1}^{N}{\left(y_{i}-\mu_{y}\right)}^{2}$$

(26) $$\sigma_{xy}=\frac{1}{N-1}\sum_{i=1}^{N}\left(x_{i}-\mu_{x}\right)\left(y_{i}-\mu_{y}\right)$$

(27) $$UQI=\frac{4\sigma_{xy}\mu_{x}\mu_{y}}{\left(\sigma_{x}^{2}+\sigma_{y}^{2}\right)\left[\mu_{x}^{2}+\mu_{y}^{2}\right]}$$

In Equation (27), the dynamic range of the *UQI* value is [−1, 1]. The optimal value is 1, which can only be achieved when the two images are identical. In the equations, *μ* represents the mean, and *σ* represents the variance. In fact, the *UQI* value is the premise of the *SSIM* calculation. The *SSIM* metric is calculated by Equation (28).

(28) $$SSIM\left(x,y\right)=\frac{\left(2\mu_{x}\mu_{y}+C_{1}\right)\left(2\sigma_{xy}+C_{2}\right)}{\left(\mu_{x}^{2}+\mu_{y}^{2}+C_{1}\right)\left(\sigma_{x}^{2}+\sigma_{y}^{2}+C_{2}\right)}$$

Comparing Equations (28) and (27), it can be observed that the difference in the equations is due to the *C*_1_ and *C*_2_ coefficients. The *UQI* value is achieved when *C*_1_ and *C*_2_ in the *SSIM* equation are both set to 0.

## 5. Numerical Results and Discussion

In this study, 1000 complex-valued backscattering electric field data were generated numerically using the setup in Figure 1, and the parameters and values in Table 1. The magnitudes of this data were used to create a dataset for real-valued neurocomputational models, while another dataset was generated for the CV-MWINet model using the original complex values along with 12 measured values. To improve the generalizability of the proposed models, the number of data was kept as high as possible. Thus, the input data have the dimensions (1000, 301, 90, 1), whereas the output data have the dimensions (1000, 512, 512, 1). To simplify training and testing of the models, the output images were resized to have dimensions (1000, 128, 128, 1). The 10-fold cross-validation method was used for the performance evaluation of the proposed neurocomputational models. Although different epochs were used to train the models, 1000 epochs and 32 batch sizes were chosen in the 10-fold cross-validation process for the four models. Table 3 provides a comparison of the evaluation results obtained through 10-fold cross-validation using the train data. The values in Table 3 are expressed as the mean value ± the standard deviation.

MSE and SSIM metrics are presented in Table 3 for the proposed RV-DNN and RV-CNN models, while ACC and SSIM metrics are presented for the MWINet models. This is because the proposed RV-DNN and RV-CNN models use float-valued output images for training, whereas the MWINet models use binary-valued output images. On examining the data in Table 3, it can be seen that the RV-DNN model has a higher MSE error than the RV-CNN model, while the SSIM metrics are greater for the RV-DNN model than for the RV-CNN model. A comparison of the 10-fold cross-validation results of the MWINet models with those of the other models indicates that the MWINet models have superior training performance. Table 4 presents a comparison of the 10-fold cross-validation performance of the proposed neurocomputational models using test data.

**Figure 7:**
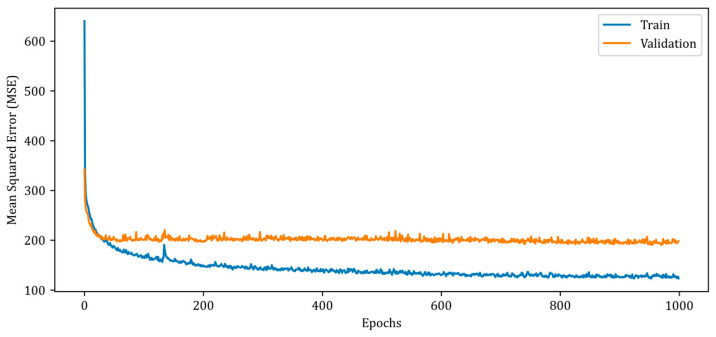
Mean squared error (MSE) curves for training and validation phases of the proposed RV-DNN model.

In terms of MSE error, the RV-CNN model outperforms the RV-DNN model, although the SSIM values are comparable. The MWINet models are observed to produce superior outcomes compared to the proposed RV-DNN and RV-CNN models. After a 10-fold cross-validation, the dataset was shuffled, and neurocomputational models were then trained utilizing 90% of the data. The remaining data was utilized for both validation and testing. Figure 7 illustrates the change in the MSE measure during the training and validation of the RV-DNN model.

**Figure 8:**
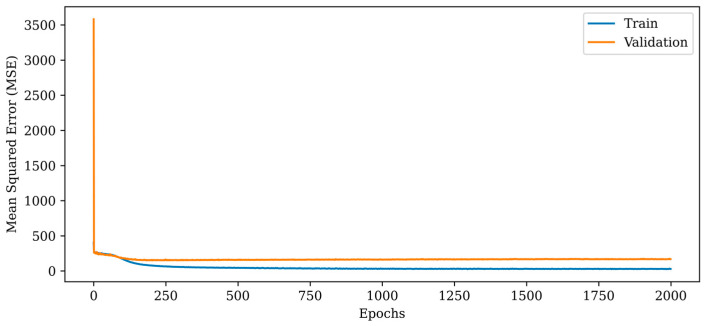
Mean squared error (MSE) curves for training and validation phases of the proposed RV-CNN model.

In Figure 7, the MSE error for the training data begins at a high value and rapidly decreases below 200 in the early epochs. However, after the 20th epoch, the rate of error reduction decreases and follows a monotonic downward trend over the thousand epochs. The validation MSE error, on the other hand, follows a monotonic trajectory of about 200, albeit with minor ripples. The MSE errors of the proposed RV-DNN model are obtained as 103.40007 and 96.39562 during training and testing, whereas the SSIM metrics are calculated as 0.92424 and 0.93020, respectively. Figure 8 depicts the change in the MSE metric during the training and validation of the proposed RV-CNN model.

The MSE metric indicates a dramatic fall in the initial epochs and a monotonic reduction in the subsequent epochs, as depicted in Figure 8. In comparison to the proposed RV-DNN model, the RV-CNN model exhibits a closer variance between the train and validation errors. The normalization layers used in the model help to keep the validation error close to the train error. The MSE errors of the proposed RV-CNN model are obtained as 45.283 and 153.818 during training and testing, whereas the SSIM metrics are calculated as 0.91000 and 0.92300, respectively.

**Figure 9:**
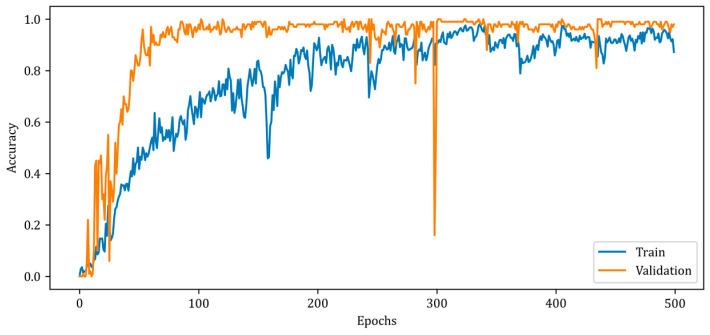
Accuracy curves for training and validation phases of the proposed RV-MWINet model.

Figure 9 depicts the change in the accuracy metric of the RV-MWINet model during training and validation.

**Figure 10:**
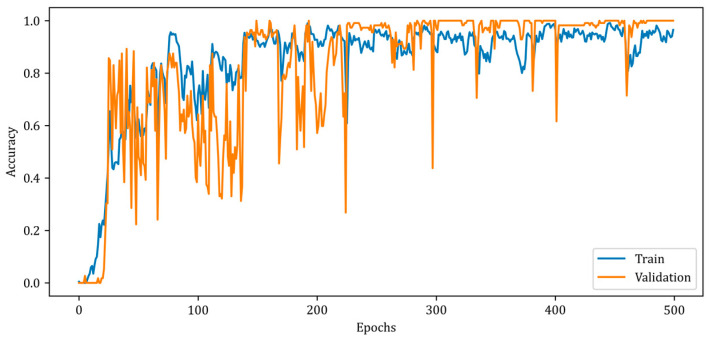
Accuracy curves for training and validation phases of the proposed CV-MWINet model.

Figure 9 illustrates a slower rise in training accuracy compared to validation accuracy. Due to the chosen batch size and the fact that the solution space has a high number of local minimums, the accuracy curves contain numerous ripples. Due to the design of the RV-MWINet model, both the CNN structure in the first model layers and the U-Net-based model layers are trained simultaneously. Since image generation and improvement are performed concurrently, it is acceptable for the number of ripples to increase throughout training and validation. The MSE, SSIM, and accuracy metrics for the training phase of the proposed RV-MWINet model are 0.00083, 0.99996, and 0.91139, while the same metrics for the testing process are 0.00467, 0.99957, and 0.86359. To account for the effect of the phase component of the complex-valued backscattered electric field data, each layer of the MWINet model in Figure 6 was replaced with a complex-valued layer to construct the CV-MWINet model structure. Figure 10 illustrates the evolution of the accuracy metrics of the proposed CV-MWINet model for training and validation over 500 epochs.

During the training of the CV-MWINet model, the complex average cross-entropy (CACE) loss function as given by Equation (29) was utilized, and the model weights at the iteration with the effective weight distribution were kept.

(29) LossACE=12[LossCCE(Re(ypred),ytrue)+LossCCE(Im(ypred),ytrue)]

In Equation (29), ACE and CCE represent average cross-entropy and category cross-entropy, respectively. The proposed CV-MWINet model was trained for 500 epochs with a batch size of 32 and achieved a training accuracy of 0.991 and a validation accuracy of 1.000.

**Figure 11:**
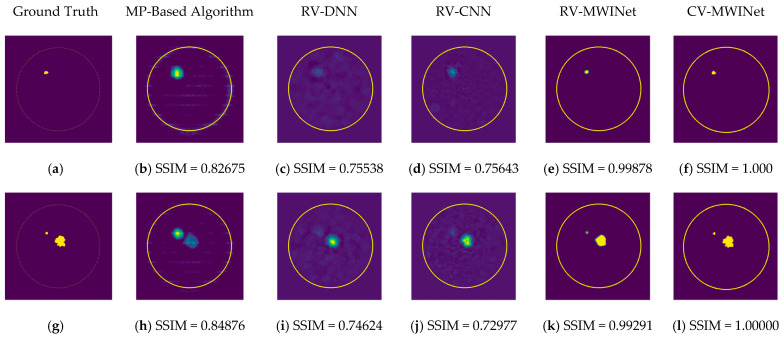

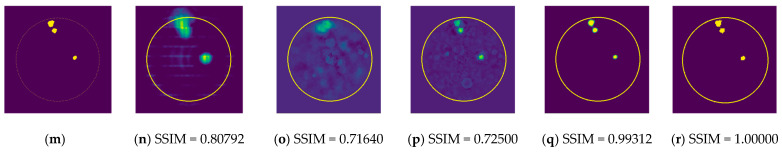
Comparison of samples of microwave images generated by the proposed neurocomputational models for train data ((**a**,**g**,**m**) are ground truth images).

In order to compare the performance of the proposed models, the RV-DNN, RV-CNN, and MWINet models are employed to generate images from data samples. Also, images were generated using the conventional MP-based MWI imaging technique using the same data. Figure 11 depicts the images generated by randomly selected training data samples. The ground truth images are depicted in Figure 11a,g,m.

**Figure 12:**
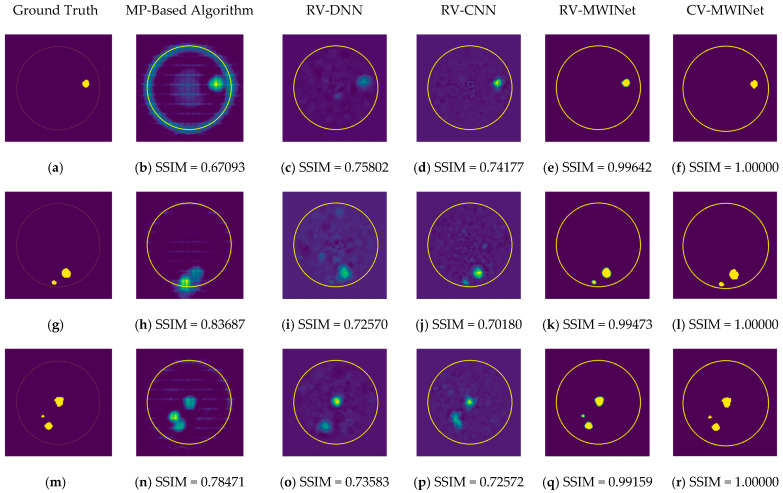
Comparison of samples of microwave images generated by the proposed neurocomputational models for test data ((**a**,**g**,**m**) are ground truth images).

Figure 11b,h,n depict radar-based images generated by the MP-based method for data containing one tumor, two tumors, and three tumors, respectively. Even though the backscattered electric field data contains information on a relatively modest scatterer, the radar-based MP-based image can make this scatterer appear larger than it actually is when MP-based images are evaluated. According to the case involving a single tumor, the image obtained from the RV-DNN model provides limited information regarding the position of the tumor. Although the RV-CNN model produces a clearer image of the same tumor, the RV-MWINet model is seen to produce the most accurate image. In cases involving two tumors, the MP-based algorithm generated a substantially larger image for the smaller tumor. In the images generated by the RV-DNN and RV-CNN models proposed, the small tumor is not visible. In this scenario, the RV-MWINet model delivers the most accurate representation of ground truth. Figure 11k demonstrates that the RV-MWINet model is able to image relatively small tumors. In the scenario involving three tumors, one tumor is positioned far away, while the other two are located quite close to one another. In this case, the MP-based algorithm treats two nearby tumors as a single tumor, as shown in Figure 11n. The RV-DNN model does not provide a good solution for distinguishing between two tumors, and the resulting image is quite noisy. The image generated by the proposed RV-CNN model is superior to those generated by the conventional method and the RV-DNN model, but it also contains noise. Figure 11q depicts the image generated by the RV-MWINet model, which is the image most similar to the ground truth. Images obtained with CV-MWINet are given in Figure 11f,l,r. When these images are analyzed, it can be observed that they are identical to the ground truth images. It may be stated that processing the complex-valued input information in complex-valued layers without losing the imaginary component of the data enhances the image quality at the output of the CV-MWINet. Similar to Figure 11, Figure 12 shows the images generated by the MP-based algorithm, RV-DNN model, RV-CNN model, and MWINet models for test data samples. In the case of a single tumor, the location of the tumor can be detected, albeit imprecisely, using blurry images obtained with the MP-based algorithm, RV-DNN model, and RV-CNN model. As seen in Figure 12e, RV-MWINet provided the cleanest and finest image for this case. In a scenario with two tumors, the MP-based method generates a rather large tumor image for the small tumor, as depicted in Figure 12h. This image also demonstrates that the MP-based algorithm depicts the tumor as being sufficiently massive to extend beyond the skin. The RV-DNN-based image in this scenario is quite noisy, so only the position of the major tumor is recognizable. Even if the image is noisy, the RV-CNN model can generate a better image than other models. In contrast, the MWINet models generated the most precise results in these scenarios. In all test scenarios, the CV-MWINet model achieves the best results compared to the other models, while the RV-MWINet model produces results that are comparable to those of the CV-MWINet model. It is possible to say that the usage of complex-valued data improves the performance of the model.

In the final scenario with two large tumors and one small tumor, the MP-based algorithm presents two adjacent tumors as if they were a single tumor. The small tumor is not visible in the image generated by the RV-DNN model. In this case, the RV-CNN model displays two adjacent tumors as a single tumor. However, as shown in Figure 12q,r, the RV-MWINet and CV-MWINet models accurately predicted the location and size of the three tumors in this scenario.

In order to analyze the results of the application of the models to a real-world problem following the simulation studies, a metal screw was placed in fine sand and a tumor phantom was placed in a healthy phantom, and measurement data was collected using a horn antenna and an Agilent vector network analyzer in accordance with the monostatic CSAR principle. The utilization of metal in fine sand allows the analysis of the effects of PEC material in a homogeneously distributed environment, whereas the tumor phantom placed within a healthy phantom is a method of simulating a realistic patient. In the measurement scenarios presented in Table 5, the scatterers were placed at a specific distance and a 45-degree angle to the *x*-axis relative to the center of the imaging domain.

**Figure 13:**
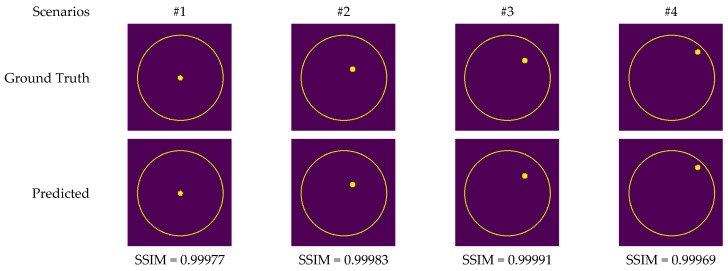
Comparison of samples of microwave images generated by the proposed CV-MWINet model for measurement data (metal screw in fine dust).

Figure 13 depicts the images generated by the CV-MWINet model using data that was collected from measurements of scenarios involving metal screws in fine sand.

**Figure 14:**
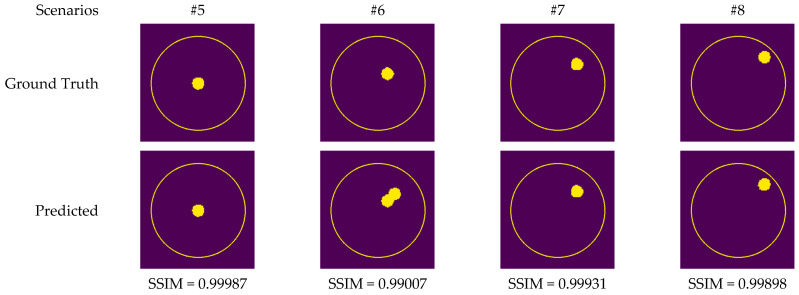
Comparison of samples of microwave images generated by the proposed CV-MWINet model for measurement data (tumor phantom in healthy phantom).

Although measurement results were also utilized to train CV-MWINet, the performance of the model for measurement data was also remarkably precise. Figure 14 illustrates images generated from the CV-MWINet model utilizing measurement data with the tumor phantom located within the healthy phantom. In scenarios utilizing phantoms, where the radius of the tumor phantom is around 2 cm, the tumors in the images are also large. Figure 14 illustrates that in scenarios #5, #7, and #8, the images obtained from the model closely match the ground truth images, however, in scenario #6, the images derived from the CV-MWINet model depict two adjacent tumors when there should be only one tumor. One of the main reasons for this inaccuracy is due to the use of a small number of measurement data in the dataset used to train the model. It can be stated that increasing the number of measurement data yields more precise results.

Table 6 provides PSNR, UQI, and SSIM metrics for the entire dataset in addition to simulation data for the models proposed in this study.

Analyzing the numerical metrics in Table 6 reveals that the neurocomputational models proposed in this study produce images of higher quality than conventional techniques. Even if the metrics of the proposed RV-DNN and RV-CNN models are comparable, it is noticeable that the RV-CNN model outperforms the RV-DNN model when analyzing images. Images and metrics provided by the MWINet models demonstrate that this model generates exceptionally high-quality microwave images. Even though their training time is longer, it is a well-known fact that deep learning models generate images quickly during the testing phase. In contrast, traditional algorithms can generate images over extended periods of time. The times required to generate the traditional images depicted in Figure 11 and Figure 12 are listed in Table 7, based on the mesh size employed by the MP-based method utilized in this study.

As shown in Table 7, imaging was carried out using an MP-based technique with 9061 and 16,105 mesh points. The imaging time required by the MP-based approach is not dependent on the number of tumors but is heavily reliant on the number of mesh points. The neurocomputational models proposed in this study can generate images of superior quality in less time than conventional techniques.

## 6. Conclusions

In this study, three distinct neurocomputational models based on DNNs, CNNs, and U-Net are presented for radar-based microwave imaging using raw backscattered electric field data. The neurocomputational models proposed in this study are trained and tested using backscattered electric field data collected via the CSAR concept. In the training and testing phases, the RV-DNN model gives results with MSE errors of 103.40007 and 96.39562, whereas the RV-CNN model produces results with 45.283 and 153.818 errors for the same data. Similarly, the RV-DNN model produced images with SSIMs of 0.92424 and 0.93020 in the training and testing phase, while the RV-CNN model produced images with SSIMs of 0.91000 and 0.92300. The MSE, SSIM, and accuracy metrics for the training phase of the proposed RV-MWINet model are 0.00083, 0.99996, and 0.91139, while the same metrics for the testing process are 0.00467, 0.99957, and 0.86359. For the CV-MWINet model using complex-valued data, PSNR, UQI, and SSIM values as training metrics were obtained as 209.09540, 0.96754, and 1.00000, respectively, while the same metrics for the test were obtained as 209.46525, 0.96995, and 1.00000, respectively. The images generated by neurocomputational models are compared to those obtained by MP-based algorithm. Analyzing the obtained images demonstrates that the proposed neurocomputational models generate more effective results.

## Figures and Tables

**Table 1:** Values for simulation parameters.

Parameter	Value
Start Frequency (GHz)	1
Stop Frequency (GHz)	10
Frequency Count	301
Skin Radius (cm)	7
Gap Between Skin and Antenna (cm)	2
Number of Tumor Scatterers	1–3
Radius Range of Tumor Scatterers (cm)	0.2–0.9
Rotation Angle Increment (°)	4

**Table 2:** Properties of the proposed CNN-based model layers.

Layer	Output Shape	Number of Parameters
Convolution 2D	(299, 89, 32)	288
Batch Normalization	(299, 89, 32)	128
Convolution 2D	(297, 86, 32)	9216
Batch Normalization	(297, 86, 32)	128
Maximum Pooling 2D	(99, 28, 32)	-
Convolution 2D	(97, 26, 64)	18,432
Batch Normalization	(97, 26, 64)	256
Convolution 2D	(95, 24, 64)	36,864
Batch Normalization	(95, 24, 64)	256
Maximum Pooling 2D	(31, 8, 64)	-
Convolution 2D	(29, 6, 128)	73,728
Batch Normalization	(29, 6, 128)	512
Convolution 2D	(27, 4, 128)	147,456
Batch Normalization	(27, 4, 128)	512
Convolution 2D	(25, 2, 128)	147,456
Batch Normalization	(25, 2, 128)	512
Flatten	6400	-
Fully Connected #1	2048	13,107,200
Batch Normalization	2048	8192
Fully Connected #2	2048	4,196,352
Fully Connected #3	16,384	33,570,816

**Table 3:** Performance metrics of the proposed neurocomputational models for 10-fold cross-validation using train data.

Parameters		RV-DNN		RV-CNN		RV-MWINet		CV-MWI-Net	
		MSE	SSIM	MSE	SSIM	ACC	SIM	ACC	SSIM
10-fold Cross-Validation	Fold #1	97.784 ± 45.153	0.918 ± 0.031	62.731 ± 33.540	0.897 ± 0.051	0.999 ± 0.001	1.000 ± 0.000	1.000 ± 0.000	1.000 ± 0.000
	Fold #2	100.917 ± 47.951	0.925 ± 0.029	75.192 ± 42.959	0.893 ± 0.052	0.988 ± 0.005	0.998 ± 0.000	1.000 ± 0.000	1.000 ± 0.000
	Fold #3	102.443 ± 48.706	0.922 ± 0.030	65.007 ± 41.774	0.888 ± 0.054	0.998 ± 0.002	1.000 ± 0.000	1.000 ± 0.000	1.000 ± 0.000
	Fold #4	92.100 ± 43.208	0.925 ± 0.029	74.251 ± 48.942	0.886 ± 0.053	0.994 ± 0.004	0.999 ± 0.000	1.000 ± 0.000	1.000 ± 0.000
	Fold #5	101.076 ± 47.054	0.924 ± 0.031	61.865 ± 38.730	0.887 ± 0.054	0.998 ±0.001	1.000 ± 0.000	1.000 ± 0.000	1.000 ± 0.000
	Fold #6	92.854 ± 41.111	0.924 ± 0.031	79.385 ± 47.123	0.890 ± 0.058	1.000 ± 0.000	1.000 ± 0.000	1.000 ± 0.000	1.000 ± 0.000
	Fold #7	98.932 ± 46.810	0.924 ± 0.030	65.930 ± 49.868	0.891 ± 0.056	0.999 ± 0.001	1.000 ± 0.000	1.000 ± 0.000	1.000 ± 0.000
	Fold #8	98.564 ± 45.503	0.925 ± 0.030	61.795 ± 39.846	0.890 ± 0.052	0.999 ± 0.001	1.000 ± 0.000	1.000 ± 0.000	1.000 ± 0.000
	Fold #9	102.653 ± 49.134	0.921 ± 0.031	61.114 ± 43.199	0.892 ± 0.053	0.994 ± 0.004	1.000 ± 0.000	1.000 ± 0.000	1.000 ± 0.000
	Fold #10	93.116 ± 43.122	0.927 ± 0.030	71.750 ± 58.493	0.888 ± 0.057	0.996 ± 0.003	1.000 ± 0.000	1.000 ± 0.000	1.000 ± 0.000
	**Average**	98.044 ± 45.775	0.924 ± 0.030	67.902 ± 44.447	0.890 ± 0.054	0.997 ± 0.002	1.000 ± 0.000	1.000 ± 0.000	1.000 ± 0.000

**Table 4:** Performance metrics of the proposed neurocomputational models for 10-fold cross-validation using test data.

Parameters		RV-DNN		RV-CNN		RV-MWINet		CV-MWINet	
		MSE	SSIM	MSE	SSIM	ACC	SSIM	ACC	SSIM
10-fold Cross-Validation	Fold #1	185.183 ± 124.598	0.914 ± 0.030	157.868 ± 108.600	0.915 ± 0.030	0.995 ± 0.004	1.000 ± 0.000	0.992 ± 0.005	0.999 ± 0.001
	Fold #2	207.658 ± 136.553	0.912 ± 0.033	162.565 ± 107.290	0.910 ± 0.033	0.987 ± 0.005	0.998 ± 0.000	0.993 ± 0.005	0.999 ± 0.001
	Fold #3	195.671 ± 132.356	0.911 ± 0.032	156.713 ± 109.397	0.910 ± 0.027	0.993 ± 0.004	0.999 ± 0.001	0.993 ± 0.004	0.999 ± 0.001
	Fold #4	200.928 ± 137.845	0.919 ± 0.027	163.380 ± 109.841	0.906 ± 0.032	0.992 ± 0.005	0.999 ± 0.001	0.993 ± 0.004	0.999 ± 0.001
	Fold #5	181.389 ± 119.239	0.916 ± 0.031	152.357 ± 107.160	0.915 ± 0.028	0.993 ± 0.004	0.999 ± 0.000	0.993 ± 0.005	0.999 ± 0.001
	Fold #6	216.705 ± 140.831	0.912 ± 0.030	178.969 ± 122.073	0.909 ± 0.033	0.993 ± 0.005	0.999 ± 0.001	0.993 ± 0.005	0.999 ± 0.001
	Fold #7	202.940 ± 135.807	0.915 ± 0.029	168.076 ± 117.404	0.910 ± 0.032	0.993 ± 0.004	0.999 ± 0.001	0.994 ± 0.004	0.999 ± 0.000
	Fold #8	187.140 ± 126.529	0.914 ± 0.024	150.096 ± 112.963	0.911 ± 0.030	0.995 ± 0.004	1.000 ± 0.000	0.994 ± 0.004	0.999 ± 0.000
	Fold #9	198.709 ± 135.104	0.914 ± 0.028	164.587 ± 112.925	0.913 ± 0.028	0.991 ± 0.006	0.999 ± 0.001	0.992 ± 0.005	0.999 ± 0.001
	Fold #10	197.682 ± 123.131	0.916 ± 0.029	166.285 ± 111.977	0.910 ± 0.029	0.993 ± 0.004	0.999 ± 0.001	0.993 ± 0.005	0.999 ± 0.001
	**Average**	197.401 ± 131.199	0.914 ± 0.030	162.089 ± 111.963	0.911 ± 0.030	0.993 ± 0.005	0.999 ± 0.001	0.993 ± 0.004	0.999 ± 0.001

**Table 5:** Scenarios used in the measurement.

Scenarios	Materials	Distance from the Center (cm)
#1	Metal screw in fine sand	0
#2		2
#3		4
#4		6
#5	Tumor phantom in healthy phantom	0
#6		2
#7		4
#8		5.5

**Table 6:** Performance metrics of the proposed neurocomputational models for the images given in Figure 9 and Figure 10.

Metrics/Models		Train Data			Test Data			Avgs. ± Stds.	
		1 Tumor	2 Tumor	3 Tumor	1 Tumor	2 Tumor	3 Tumor	All Train Set	All Test Set
**PSNR (dB)**	MP-Based Algorithm	25.87656	24.14579	22.83756	19.78088	23.12839	22.51522	–	–
	RV-DNN Model	23.0948	22.02725	17.7845	23.76579	18.924	21.27754	20.37510 ± 2.89746	20.52958 ± 2.93180
	RV-CNN Model	23.62187	22.1329	19.95046	21.513	20.54329	19.71726	21.22355 ± 2.27647	21.38717 ± 2.62633
	RV-MWINet Model	42.35213	34.39235	34.32751	37.00931	35.94595	34.00697	34.68058 ± 3.24353	34.57853 ± 3.53797
	CV-MWINet Model	217.02188	207.71069	209.097967	210.92949	207.84857	206.52970	209.09540 ± 3.56411	209.46525 ± 3.59434
**UQI**	MP-based Algorithm	0.914	0.92553	0.90138	0.82	0.91924	0.89136	–	–
	RV-DNN Model	0.74023	0.73941	0.71659	0.74554	0.72334	0.73738	0.72929 ± 0.01239	0.72974 ± 0.1239
	RV-CNN Model	0.74212	0.73895	0.72828	0.73449	0.73098	0.72887	0.73380 ± 0.00854	0.73426 ± 0.00957
	RV-MWINet Model	0.9995	0.99792	0.99783	0.9986	0.99842	0.99758	0.99759 ± 0.00172	0.99750 ± 0.00211
	CV-MWINet Model	0.99118	0.967916	0.966361	0.98312	0.96825	0.95368	0.96754 ± 0.01632	0.96995 ± 0.01479
**SSIM**	MP- Based Algorithm	0.82675	0.84876	0.80792	0.67093	0.83687	0.78471	–	–
	RV-DNN Model	0.75538	0.74624	0.7142	0.75802	0.7257	0.73583	0.73705 ± 0.02006	0.73754 ± 0.01913
	RV-CNN Model	0.75643	0.72977	0.725	0.74177	0.7018	0.72572	0.73220 ± 0.01953	0.73457 ± 0.02198
	RV-MWINet Model	0.99878	0.99291	0.99312	0.99642	0.99473	0.99159	0.99295 ± 0.00396	0.99302 ± 0.00419
	CV-MWINet Model	1.00000	1.00000	1.00000	1.00000	1.00000	1.00000	1.00000 ± 0.00000	1.00000 ± 0.00000

– Not available.

**Table 7:** Image generation time with the MP-based algorithm.

Mesh Points		9061 Points	16,105 Points
Train Data	1 Tumor	189.96657 s	385.11506 s
	2 Tumor	186.09587 s	391.25924 s
	3 Tumor	184.26689 s	337.86427 s
Test Data	1 Tumor	180.65272 s	386.98390 s
	2 Tumor	185.19212 s	370.29391 s
	3 Tumor	184.13824 s	376.40420 s
**Avgs. ± Stds.**		185.05210 ± 3.03536 s	374.6534 ± 19.55980 s

## Data Availability

The data presented in this study are available on request from the corresponding author.
